# Supplementary material for: Evolutionary transition from a single RNA replicator to a multiple replicator network
Source: Nat Commun. 2022 Mar 18;13:1460. doi: 10.1038/s41467-022-29113-x (PMC8933500; doi:10.1038/s41467-022-29113-x)
Supplement: Supplementary file 3 — Description of Additional Supplementary Files [file 41467_2022_29113_MOESM3_ESM.pdf]

Description of Additional Supplementary Files

File Name: Supplementary Data 1
Description: The RNA sequences of the isolated clones and information on all analyzed genotypes.
