# Supplementary material for: Evolutionary transition from a single RNA replicator to a multiple replicator network
Source: Nat Commun. 2022 Mar 18;13:1460. doi: 10.1038/s41467-022-29113-x (PMC8933500; doi:10.1038/s41467-022-29113-x)
Supplement: Supplementary file 5 — Reporting Summary [file 41467_2022_29113_MOESM5_ESM.pdf]

nature portfolio

Corresponding author(s): Ryo Mizuuchi, Norikazu Ichihashi

Last updated by author(s): Feb 4, 2022

Reporting Summary

Nature Portfolio wishes to improve the reproducibility of the work that we publish. This form provides structure for consistency and transparency in reporting. For further information on Nature Portfolio policies, see our Editorial Policies and the Editorial Policy Checklist.

Statistics

For all statistical analyses, confirm that the following items are present in the figure legend, table legend, main text, or Methods section.

n/a	Confirmed	
☐	☒	The exact sample size (n) for each experimental group/condition, given as a discrete number and unit of measurement
☐	☒	A statement on whether measurements were taken from distinct samples or whether the same sample was measured repeatedly
☒	☐	The statistical test(s) used AND whether they are one- or two-sided<br>*Only common tests should be described solely by name; describe more complex techniques in the Methods section.*
☒	☐	A description of all covariates tested
☒	☐	A description of any assumptions or corrections, such as tests of normality and adjustment for multiple comparisons
☐	☒	A full description of the statistical parameters including central tendency (e.g. means) or other basic estimates (e.g. regression coefficient) AND variation (e.g. standard deviation) or associated estimates of uncertainty (e.g. confidence intervals)
☒	☐	For null hypothesis testing, the test statistic (e.g. F, t, r) with confidence intervals, effect sizes, degrees of freedom and P value noted<br>*Give P values as exact values whenever suitable.*
☒	☐	For Bayesian analysis, information on the choice of priors and Markov chain Monte Carlo settings
☒	☐	For hierarchical and complex designs, identification of the appropriate level for tests and full reporting of outcomes
☒	☐	Estimates of effect sizes (e.g. Cohen's d, Pearson's r), indicating how they were calculated

Our web collection on statistics for biologists contains articles on many of the points above.

Software and code

Policy information about availability of computer code

Data collection	Mx3005P Real-Time PCR System, QuantStudio 3, FUSION-SL4, PacBio RS II and Sequel, ÄKTAexplorer, MAFFT v7.450, MEGA X, iTOL, Vienna RNAfold webserver (http://rna.tbi.univie.ac.at/), Python 3.7.4. (https://git.io/JcliB)
Data analysis	Python 3.7.4., ImageJ 1.52a

For manuscripts utilizing custom algorithms or software that are central to the research but not yet described in published literature, software must be made available to editors and reviewers. We strongly encourage code deposition in a community repository (e.g. GitHub). See the Nature Portfolio guidelines for submitting code & software for further information.

Data

Policy information about availability of data

All manuscripts must include a data availability statement. This statement should provide the following information, where applicable:

- Accession codes, unique identifiers, or web links for publicly available datasets
- A description of any restrictions on data availability
- For clinical datasets or third party data, please ensure that the statement adheres to our policy

Source data are provided with this paper. Supplementary Data 1 contains the RNA sequences of the isolated clones and information on all analyzed genotypes. PacBio sequence data before alignment are available on Dryad (https://doi.org/10.5061/dryad.95x69p8mr).

Field-specific reporting

Please select the one below that is the best fit for your research. If you are not sure, read the appropriate sections before making your selection.

☒ Life sciences ☐ Behavioural & social sciences ☐ Ecological, evolutionary & environmental sciences

For a reference copy of the document with all sections, see nature.com/documents/nr-reporting-summary-flat.pdf

Life sciences study design

All studies must disclose on these points even when the disclosure is negative.

Sample size	The number of reads used in the sequence analyses was described in Supplementary Table S1; randomly chosen 10000 reads were used for each round if available, and otherwise all available reads were used. Biochemical assays to support the main conclusions were performed at least three times. Transfer experiments to support the main conclusions were performed at least twice. Each simulation was performed three times. These sample sizes were determined based on previous experience and published data. No statistical methods were used to predetermine sample sizes.
Data exclusions	From the sequence data of each long-term replication experiment, we excluded minor mutations that have never reached at least 10% frequencies in the population to minimize random errors derived from error-prone replication of Qβ replicase and PacBio sequencing. If organism-derived (unaligned) sequences were found in randomly sampled reads, they were removed before further analysis (usually zero or one read per round).
Replication	All attempts at replication were successful. Biochemical assays and transfer experiments to support the main conclusions were performed at least three times and twice, respectively. Each simulation was performed three times.
Randomization	Not relevant to this study because samples were not allocated into groups.
Blinding	Not relevant to this study because samples were not allocated into groups.

Reporting for specific materials, systems and methods

We require information from authors about some types of materials, experimental systems and methods used in many studies. Here, indicate whether each material, system or method listed is relevant to your study. If you are not sure if a list item applies to your research, read the appropriate section before selecting a response.

Materials & experimental systems

n/a	Involved in the study	
☒	☐	Antibodies
☒	☐	Eukaryotic cell lines
☒	☐	Palaeontology and archaeology
☒	☐	Animals and other organisms
☒	☐	Human research participants
☒	☐	Clinical data
☒	☐	Dual use research of concern

Methods

n/a	Involved in the study	
☒	☐	ChIP-seq
☒	☐	Flow cytometry
☒	☐	MRI-based neuroimaging
